# Comparative analysis of scheduling algorithms for radio resource allocation in future communication networks

**DOI:** 10.7717/peerj-cs.546

**Published:** 2021-05-18

**Authors:** Khuram Ashfaq, Ghazanfar Ali Safdar, Masood Ur-Rehman

**Affiliations:** 1 School of Computer Science and Technology, University of Bedfordshire, Luton, England, United Kingdom; 2 James Watt School of Engineering, University of Glasgow, Glasgow, Scotland, United Kingdom

**Keywords:** Scheduling Algorithms, Radio Resource Allocation, Cellular Networks, Quality of Service

## Abstract

**Background:**

Wireless links are fast becoming the key communication mode. However, as compared to the wired link, their characteristics make the traffic prone to time- and location-dependent signal attenuation, noise, fading, and interference that result in time varying channel capacities and link error rate. Scheduling algorithms play an important role in wireless links to guarantee quality of service (QoS) parameters such as throughput, delay, jitter, fairness and packet loss rate. The scheduler has vital importance in current as well as future cellular communications since it assigns resource block (RB) to different users for transmission. Scheduling algorithm makes a decision based on the information of link state, number of sessions, reserved rates and status of the session queues. The information required by a scheduler implemented in the base station can easily be collected from the downlink transmission.

**Methods:**

This paper reflects on the importance of schedulers for future wireless communications taking LTE-A networks as a case study. It compares the performance of four well-known scheduling algorithms including round robin (RR), best channel quality indicator (BCQI), proportional fair (PF), and fractional frequency reuse (FFR). The performance of these four algorithms is evaluated in terms of throughput, fairness index, spectral efficiency and overall effectiveness. System level simulations have been performed using a MATLAB based LTE-A Vienna downlink simulator.

**Results:**

The results show that the FFR scheduler is the best performer among the four tested algorithms. It also exhibits flexibility and adaptability for radio resource assignment.

## Introduction

Wireless networks have seen a massive growth in recent years. Their popularity has surpassed wired links mainly due to cost-effective terminals and higher data rates. Guaranteeing better quality-of-service (QoS) is a necessity in today’s wireless communications as most of the multimedia applications require higher data rates. This demands the development of powerful resource management strategies that not only provide high throughput but also ensure effective usage of available resources. Scheduling algorithms are of vital importance in current as well as future wireless networks to efficiently and fairly administer the available resources (Moosavi et al., 2010). These algorithms are classified into two categories of centralized and distributed algorithms (Yazdani & Mirjalily, 2017). The centralized algorithms are typically used in downlink transmission to aptly fulfill the fairness and QoS requirements. Contrarily, the distributed algorithms are mostly employed in adhoc or uplink transmission where user can control the channel access. In this paper, four well-known scheduling algorithms are discussed, and their performance is compared considering different operating scenarios.

A scheduler must be equipped with certain components to ensure the adeptness of a wireless link. The algorithm should adequately utilize the available channel and not assign a transmission time interval (TTI) to a session with bad link quality. The algorithm should provide guaranteed short-term throughputs for error-free sessions while having long-term throughputs for all the sessions (Yazdani & Mirjalily, 2017). The redistribution of available resources among cellular users should be fairer through short-term fairness for error-free sessions and long-term fairness for error-prone sessions. The fairness index is commonly referred to the maximum difference between the normalized service received by any two backlogged sessions. Another challenge for a scheduler is to minimize the energy consumption of the cellular user. This can be achieved by allowing cellular user to transmit and receive in contiguous time slots and then go into sleep mode when idle for reduced energy consumption (Lee et al., 2015). The scheduler also needs to eliminate excessive interference by limiting the number of simultaneous transmissions in the network. In delay-sensitive applications, the scheduler should be able to provide delay bound guarantees for individual sessions (Moosavi et al., 2010). Scalability of the scheduler is another important element. The scheduler should adopt and ensure effective sharing of the available channel with increasing number of. The scheduler is also ought to be implemented with low complexity as in high-speed networks, rapid scheduling decisions are of immense importance.

Following the introduction in this section, the rest of the paper is organized as follows. First, the four key scheduling algorithms are briefly discussed followed by the review of related work including state-of-the-art. Then, details of the system model are presented. Next, a comparative study on the performance of the four schedulers is carried out using key performance metrics before the paper is concluded.

### Scheduling algorithms

The schedulers are in-charge of allocating resource blocks (RB) among cellular users according to their demand. The schedulers are primarily functioning at Evolved Node B (eNodeB) and assign both uplink and downlink resources. Based on the methodology of a scheduling algorithm, it allocates the shared resources to each cellular user at every transmission time interval (TTI). The eNodeB periodically receives channel quality indicator (CQI) from the cellular users. The higher this indicator goes, the better the channel is. Four of the key scheduling algorithms are round robin (RR), best channel quality indicator (BCQI), proportional fair (PF), and fractional frequency reuse (FFR). This section presents a brief overview of the working of these algorithms.

### Round robin (RR)

**Figure 1:**
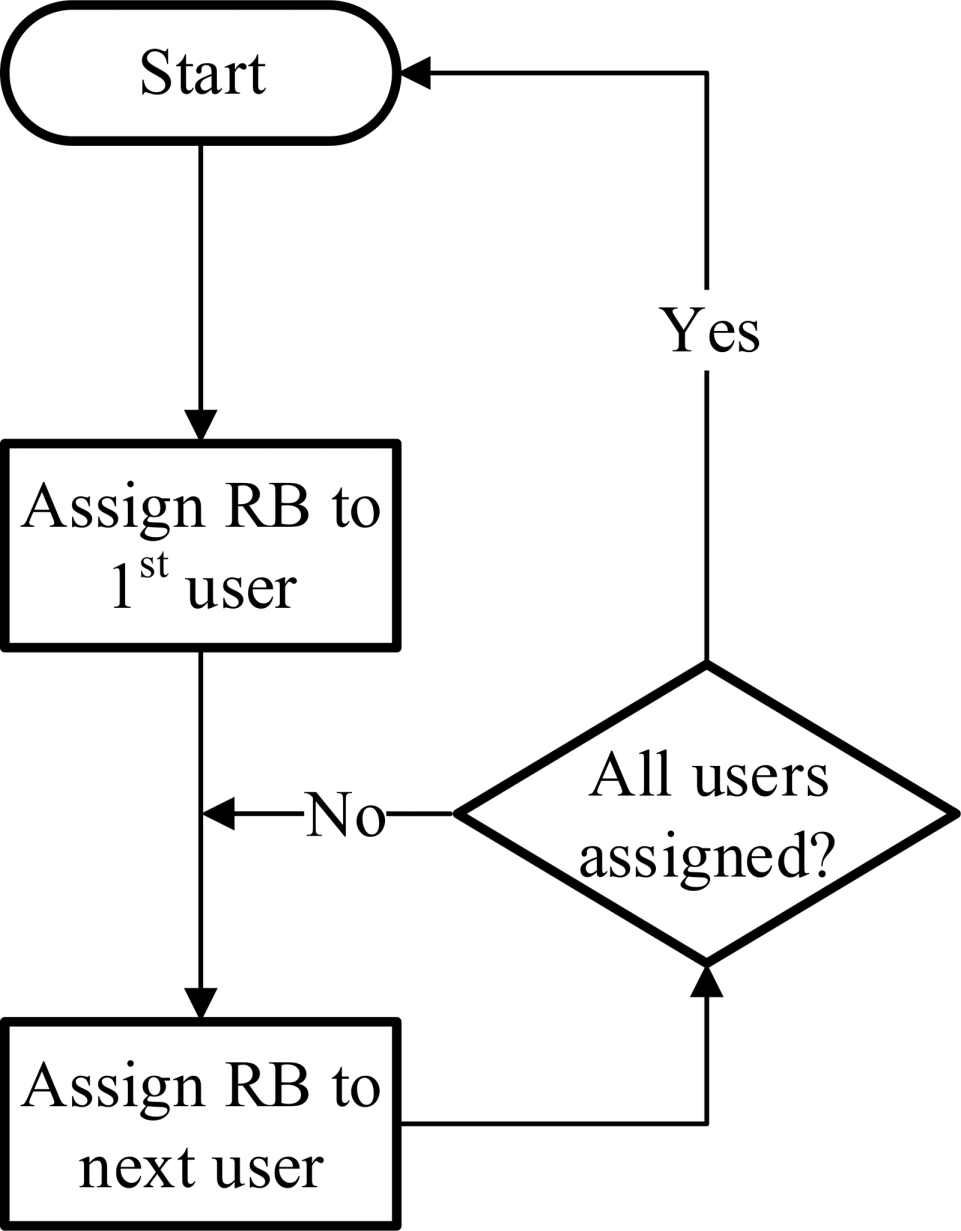
Round robin scheduler.

A round robin scheduler is a non-aware scheduling algorithm that assigns resources to every user starting from the first one and assigning resources from there on recursively. It doesn’t take into account the instantaneous channel condition (Habaebi et al., 2013). Therefore, it provides higher fairness among the users at the expense of performance. It degrades the overall system throughput as users can be assigned fading channels. Also, management of the total amount of resources is not efficient as it is not taking into account CQI factor. The reason this scheduler is widely used by many systems is the ease of implementation (Mamane et al., 2019). The basic decision-making process of this scheduler is illustrated in Fig. 1.

### Best channel quality indicator (BCQI)

**Figure 2:**
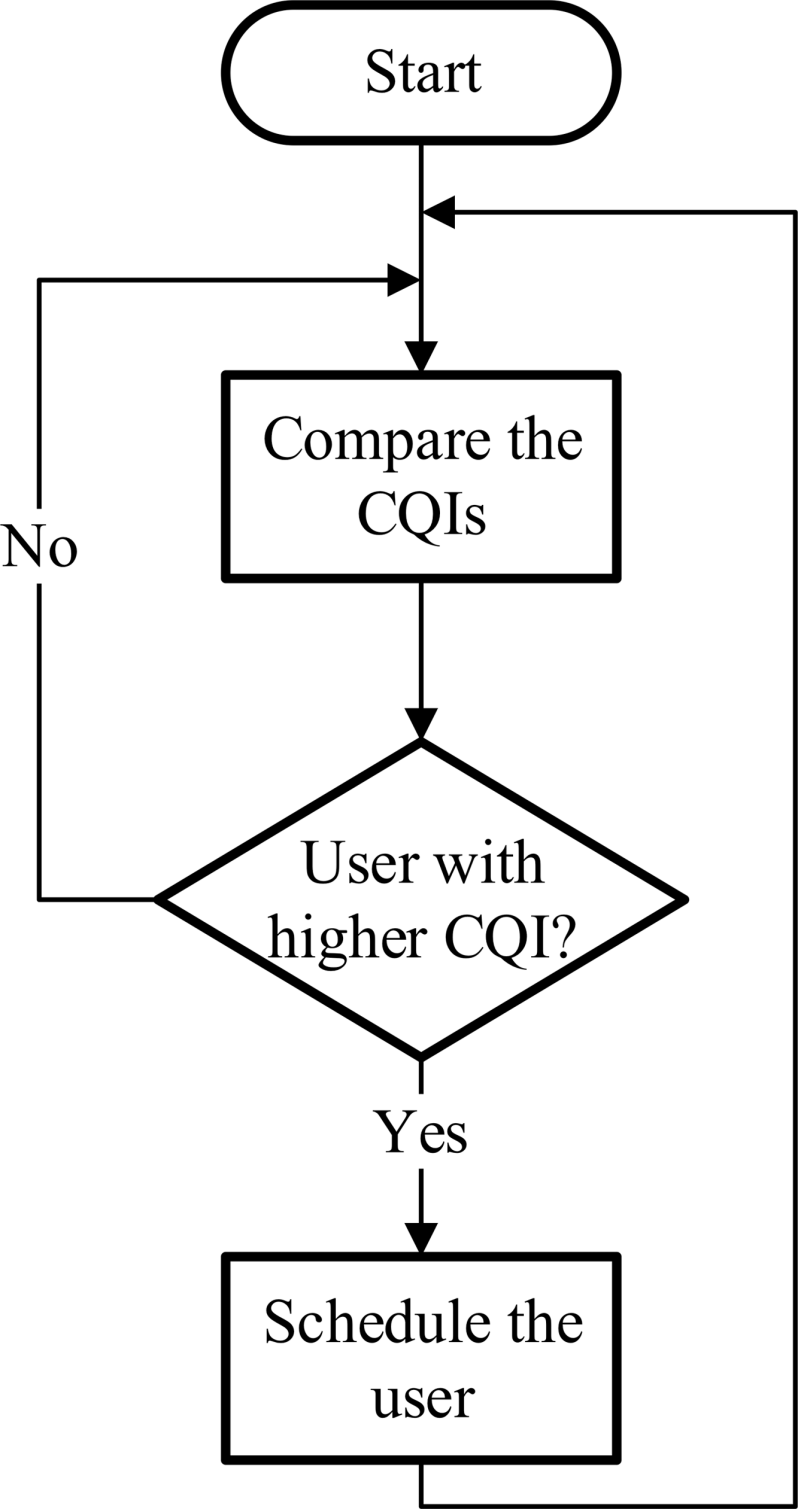
Best CQI scheduler.

The best CQI scheduler assigns resource blocks to the user with the best radio link conditions or channel quality for a particular RB at every TTI. Each cellular user sends a CQI to the eNodeB to perform the scheduling. The eNodeB transmits a reference signal (i.e., downlink pilot) in the downlink channel to the cellular user (Habaebi et al., 2013). The cellular user receives this reference signal and measures their specific CQI. The higher the CQI value the better the channel condition is. Although this scheduler can increase the individual cell throughput, it suffers from the lack of fairness among cellular users (Mamane et al., 2019). The cellular users that are located far from the base station (normally at the edge of the cell) would most likely never be scheduled. The decision-making process of this scheduler is shown in Fig. 2.

### Fractional frequency reuse (FFR)

**Figure 3:**
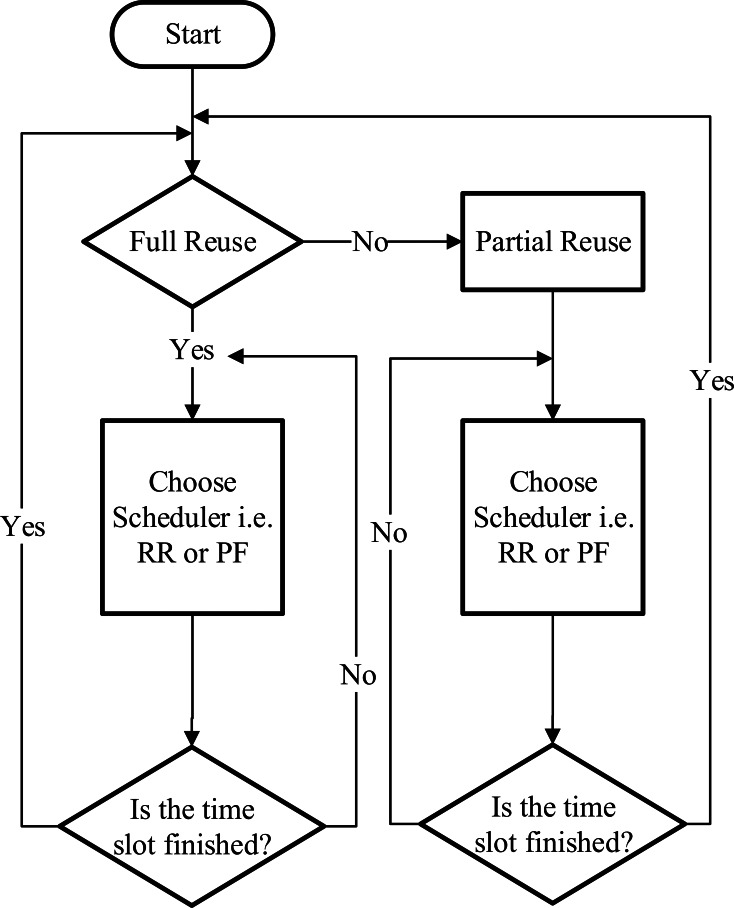
Fractional frequency reuse scheduler.

The FFR scheduler is widely implemented in scenarios where interference between adjacent cells needs to be reduced (Capozzi et al., 2013). This scheduler is also popular among research community because it allows the flexibility and adaptability to design and embed other schedulers in its process. It offers resilience to share the resource blocks among the users either in full reuse (FR) or partial reuse (PR) mode. Furthermore, available frequency band can also be sub-divided into different bands and RB can be allocated on the basis of defined priority and regions. Although FFR provides better average throughput, it lacks in fairness among users when some users are prioritized. Figure 3 illustrates the decision-making process of this scheduler.

### Proportional fair (PF)

**Figure 4:**
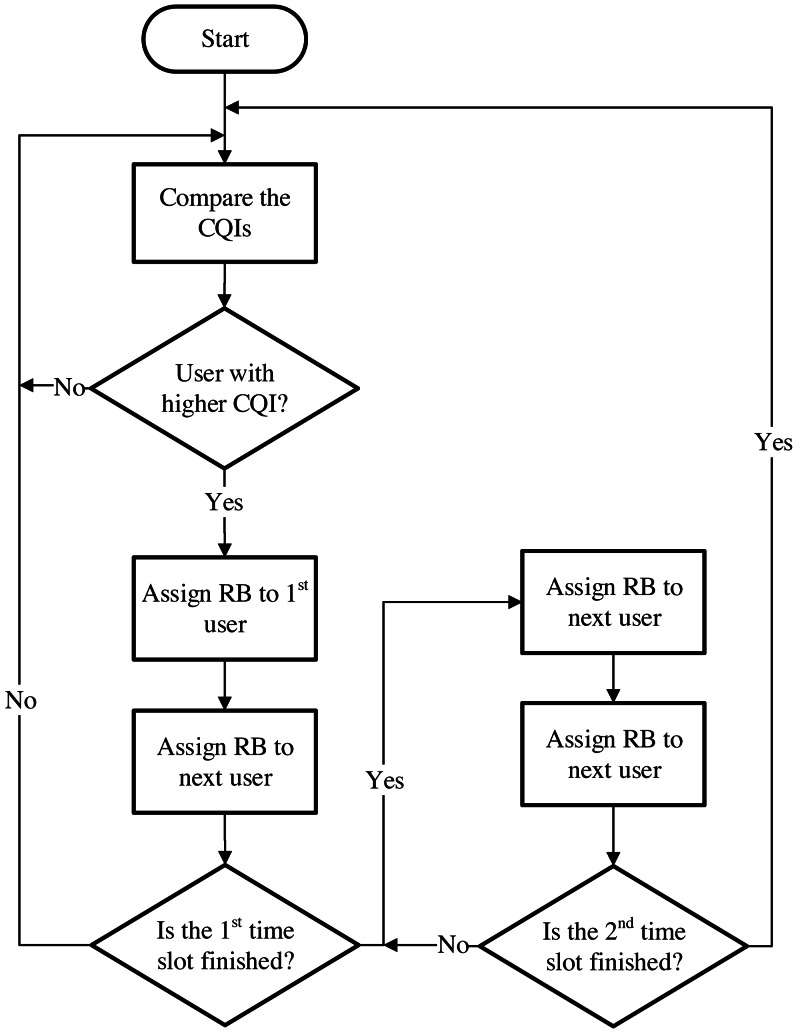
Proportional fair scheduler.

PF improves spectral efficiency and provides higher fairness to the system by using the channel variations. This scheduler allocates the resource blocks to the cellular users with the best link quality by combining CQI & level of fairness. It also allows the mobile users to achieve a maximum QoS because it maintains a balance between fairness and maximum cell throughput (Mamane et al., 2019). It allocates the RB by considering the throughput at a specific TTI and the average throughput of the cellular user. This is achieved by means of a weighted fair queueing algorithm (WFQ) that sets scheduling weights for data flow. Figure 4 depicts the decision-making process of this scheduler.

## Related Work

The study of scheduling algorithms has received substantial interest of the researchers. The authors in Habaebi et al. (2013) have presented a comparison of three scheduling algorithms namely: RR, best CQI and PF schedulers. The scheduling algorithms’ performances on the downlink were measured in terms of throughput and block error rate (BER) using a MATLAB-based system level simulator. The results have indicated that the best CQI algorithm performed better than others in terms of throughput levels but on the expense of fairness to other users suffering from bad channel conditions. In Capozzi et al. (2013), the authors have provided an extensive survey of downlink packet allocation strategies for LTE networks. The radio resource management (RRM) task was performed by the packet scheduler, which distributes radio resources among users in an efficient manner by taking into account variable channel conditions. The paper has compared scheduling algorithms with particular focus on QoS provision. In Abu-Ali et al. (2014), a detailed survey of LTE uplink schedulers is presented. The schedulers were categorized into three types: best-effort schedulers, QoS-based schedulers and power-optimizing schedulers. The system model was simulated using MATLAB and different schedulers were compared. In Zavyalova (2015), the authors have developed a simulation model for the LTE system throughput for RR and best-CQI scheduling algorithms against the input load intensity.

Small cell network (ScNet) and heterogeneous network (HetNet) have been compared for downlink performance metrics of average UE throughput, cell edge throughput and spectral efficiency in Shams, Abied & Hossain (2016). The authors have concluded that average UE throughput under HetNet is higher than ScNet making ScNet a preferred choice in densely populated areas. In Subramanian, Sandrasegaran & Kong (2016), the performance of packet scheduling (PS) algorithms of PF, maximum largest weighted delay first (MLWDF) and exponential/proportional fair (EXP/PF) has been compared using throughput, packet loss ratio (PLR), delay and fairness. The simulation results have shown that the overall system gain was increased by adding Pico cells as it provides better coverage for the edge users and increases the capacity of the network to provide better Quality of Service (QoS).

In Mahdi, Ali Yahiya & Kirci (2019), the authors have investigated variable packet scheduling algorithms of weighted fair queuing (WFQ), priority queuing (PQ) and first-in-first-out (FIFO) for the provision of best QoS in different real-time and non-real-time applications. The simulation results have shown the impact of the behavior of stationary and mobile users on QoS. In Thienthong et al. (2019), RR, PF, best-CQI, maximum throughput (Max-TP) and resource fairness (RF) schedulers were examined. This work has also considered the system embedded with cell range expansion (CRE) and almost blank subframe (ABS) mechanism to increase overall system performance while keeping low interference towards the cell edge users. The simulation results have shown that higher CRE values achieves higher edge user throughput, whereas lower CRE value results in higher peak and average throughput.

This paper extends the study presented in Habaebi et al. (2013) by comparing a larger number of schedulers with additional performance metrics. Apart from the performance metrics in the existing literature, three new parameters are employed to evaluate the schedulers; 1) mean RB occupancy; 2) peak UE throughput; and 3) edge UE throughput. To the best of our knowledge, such a detailed comparison of these scheduling algorithms is a first attempt of its kind. A MATLAB based Vienna LTE-A Downlink System Level Simulator is used for system model implementation. This simulator allows high levels of flexibility with ease of usage and scenario replication.

## System Model

A system level (SL) simulator is a powerful tool that enables study of different schedulers and their performance evaluation based on key network parameters such as throughput, latency, coverage and spectral efficiency (Shahid & Jonathan, 2014; Rupp, Schwarz & Taranetz, 2016; Jameel et al., 2018; Li et al., 2011; Cho et al., 2017; https://www.nt.tuwien.ac.at/research/mobile-communications/vccs/vienna-lte-a-simulators/lte-advanced-link-level-simulator/). The simulators also replicate rapid prototyping of novel methods and features like resource allocation and mobility management. In this work, the downlink scheduling algorithms are compared against different performance metrics using the MATLAB based Vienna LTE-A downlink system level simulator. The simulator is developed and made available by Vienna University under License Agreement for “Academic Usage” [19]. In this work, ‘Claussen’ model for shadow fading and ‘QuaDRiGa’ channel model for microscale fading is used. QuaDRiGa (Quasi Deterministic Radio Channel Generator) is a reference model in MATLAB and available as open source. It is used to depict the real time environment in system-level simulator. The main system level parameters employed in the simulation are summarized in Table 1.

**Table 1:** System parameters.

Parameter	Values
Frequency	2.14 GHz
Bandwidth	20 MHz
No of RB	100
Receiver noise figure	9 dB
No of Tx antenna ports	4
No of Rx antenna ports	4
Transmission modes	Closed Loop Spatial Multiplexing (CLSM)
Simulation Length	100 TTI
Feedback Delay	3 TTI
Inter eNodeB distance	500 m
Shadow fading type	‘None’, ‘claussen’
Microscale fading	‘None’, ‘QuaDRiGa’
Microscale pathloss	TS36942
eNodeB count	7
No of Sectors per eNodeB	3 (Total 21)
UE count	150
eNodeB tx power	40 dBm
UE distribution	Constant UEs per ROI
eNodeB antenna pattern	‘berger’
eNodeB antenna gain	15 dBi, LTE antenna, urban area (2000 MHz)
Scheduler	Best CQI, Round Robin, Proportional Fair and FFR

## Performance Evaluation and Comparative Analysis

**Figure 5:**
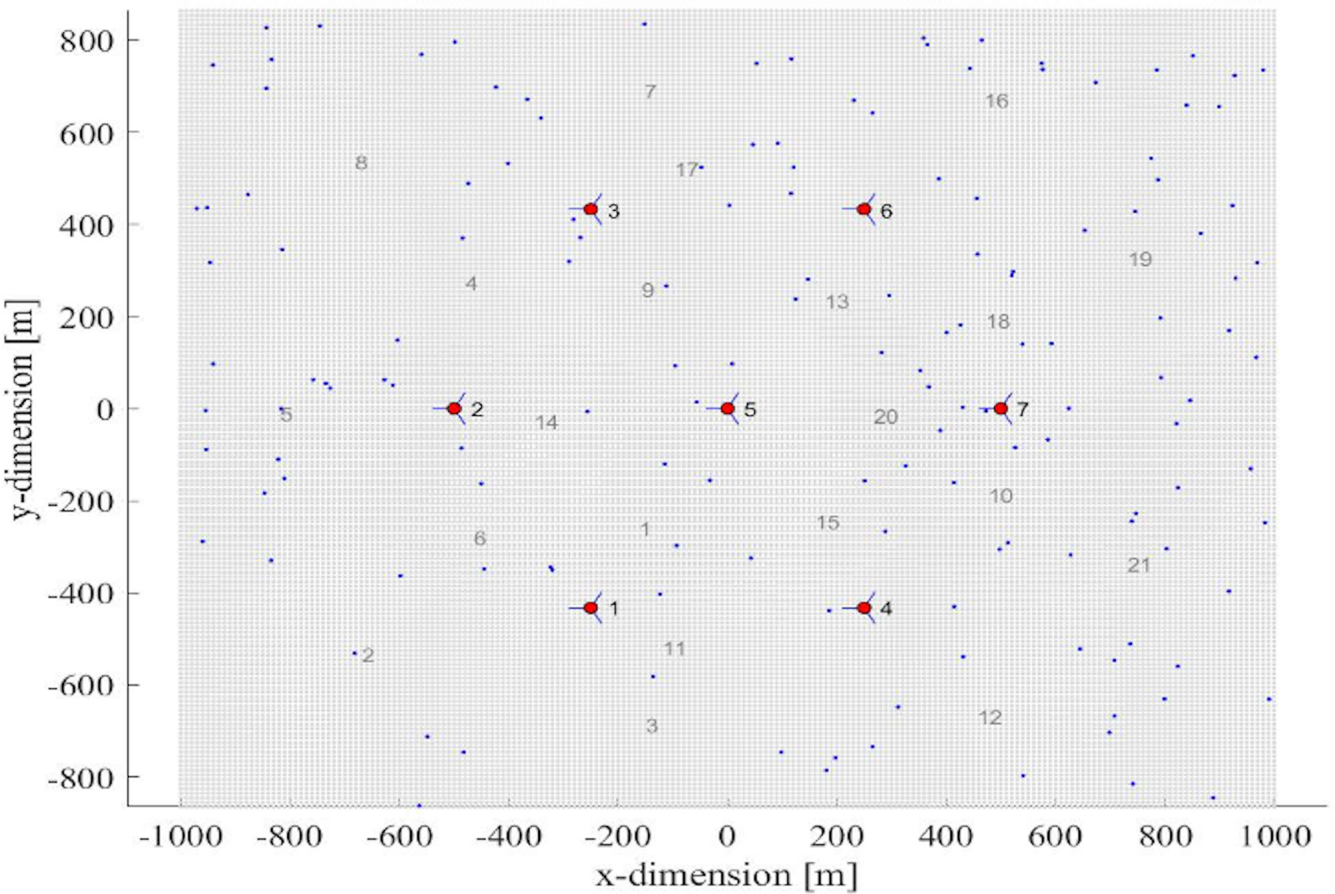
(X,Y) position of 150 UE in 21 sectors with seven eNodeB (meters).

Figure 5 shows the network layout where the red dots represent the eNodeB while the numbers represent their corresponding sector/cell. Seven eNodeBs and 21 sectors/cells are used in the simulation. The blue dots represent cellular users. The simulation has generated 150 user equipment (UEs) that are randomly distributed in the region of interest (ROI). The simulation runtime is 100 TTI (s) for each of the two considered scenarios i.e., scenario-1 without shadow fading and microscale fading channel model and scenario-2 with shadow fading (Claussen) and microscale fading (channel model QuaDRiGa). The simulations results are then compared to analyses the performance of the four schedulers.

Table 2 gives a comprehensive comparison between schedulers in terms of cell throughput, UE throughput, spectral efficiency, fairness index and RB utilization.

**Figure 6:**
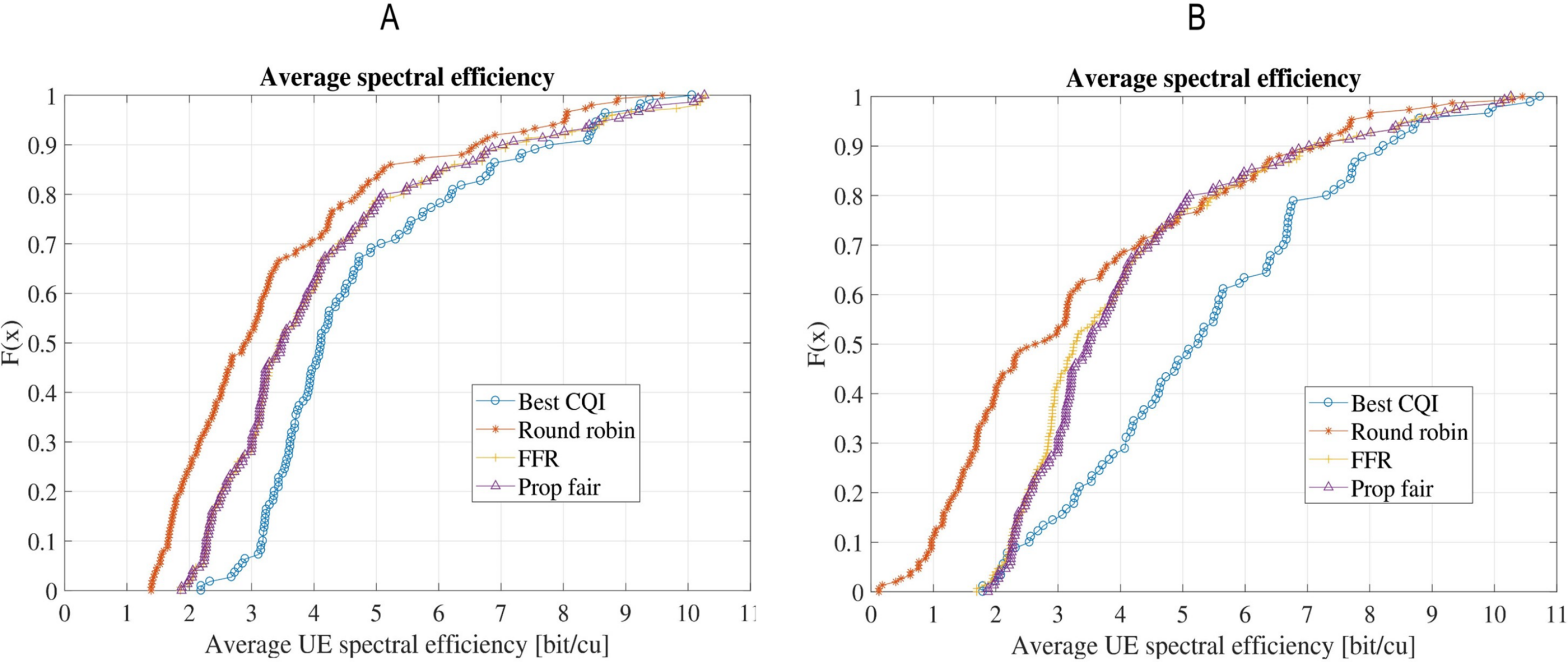
Comparison of average spectral efficiency for the four schedulers: (A) Scenario-1 without shadow/microscale fading channel model; (B) Scenario-2 with shadow (Claussen) and microscale fading (QuaDRiGa).

The spectral efficiency of the schedulers is compared in Fig. 6. Spectral efficiency refers to the information rate that can be transmitted over a given bandwidth. It is evident from these results that BCQI is the worst performer in both scenarios. It is because of the fact that BCQI only allocates the resource if the quality of the channel is good, hence, depriving the users near the cell edges with poor channel conditions. It is interesting to note that in both the scenarios, FFR and PR perform better than RR scheduler due to the higher average throughput. FFR and PF also perform equally well from spectral efficiency point of view but FFR is the recommended option due to its higher fairness index.

**Figure 7:**
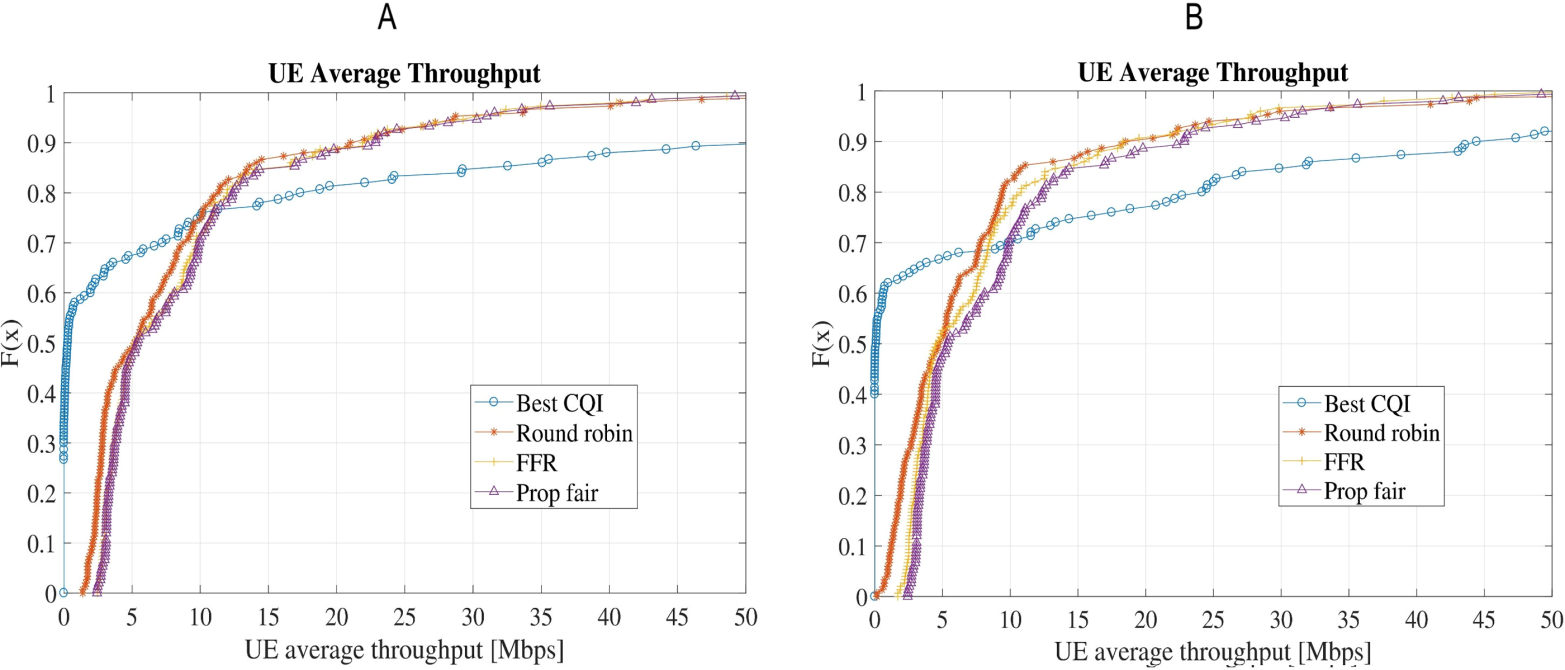
Comparison of UE average throughput for the four schedulers: (A) Scenario-1 without shadow/microscale fading channel model; (B) Scenario-2 with shadow (Claussen) and microscale fading (QuaDRiGa).

Figure 7 compares the UE’s average throughput for the four schedulers. In terms of maximum throughput, the BCQI provides a peak throughput of above 100 Mbps in both the scenarios. It makes the average throughput of BCQI looking much better than others; however, it performs the worst for the edge users due to its disposal of the user with bad channel quality. This also leads to the poorest fairness index for the BCQI among all the schedulers. FFR, PF and RR schedulers perform fairly well for average throughput but FFR outplay others with better throughput for edge users. PF has the best fairness index among all the schedulers but the peak throughput and throughput for edge user is slightly lower than that of the FFR.

**Figure 8:**
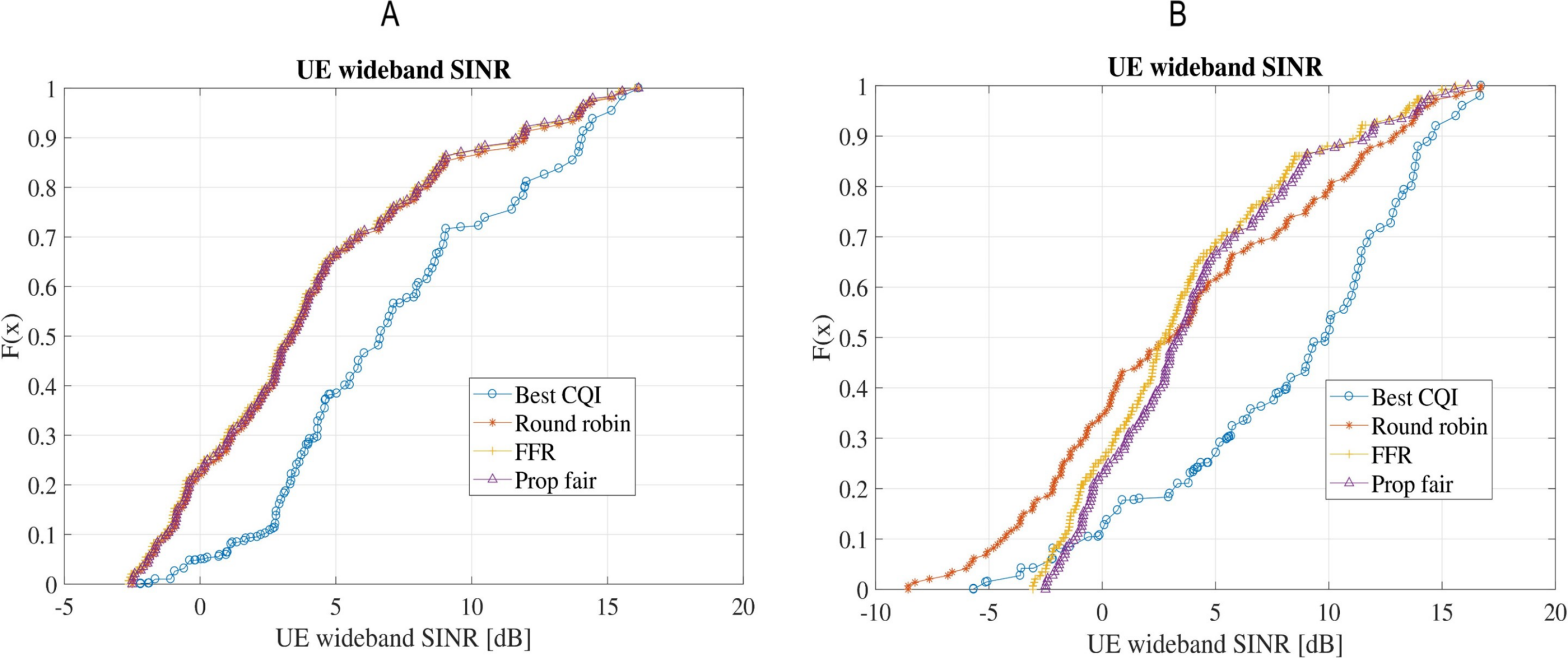
Comparison of wideband SINR for the four schedulers: (A) Scenario-1 without shadow/microscale fading channel model; (B) Scenario-2 with shadow (Claussen) and microscale fading (QuaDRiGa).

The calculated SINR of the four schedulers is compared in Fig. 8. The performance of BCQI is the worst among the four as it only transmits on the best quality channel with higher SINR. Performance of FFR, PF and RR is even considering this parameter.

**Table 2:** Performance analysis of the four key schedulers.

Parameter/Scheduler	Scenario-1 without channel model				Scenario-2 with channel model			
	RR	FFR	BCQI	PF	RR	FFR	BCQI	PF
Average UE Spectral efficiency (bit/cu)	3.46	4.17	4.80	4.15	3.38	4.21	5.37	4.21
Average cell throughput (Mb/s)	62.21	66.79	105.72	68.67	58.28	60.76	106.95	66.40
Peak UE throughput (Mbps)	28.72	30.24	102.86	31.00	29.00	27.76	95.81	8.73
Average UE throughput (Mbps)	8.71	9.35	14.80	9.61	8.16	8.51	14.97	3.10
Edge UE throughput (Mbps)	1.74	2.76	0	2.83	0.96	2.33	0	0.96
Average RBs/TTI/UE (RBs)	14.00	13.58	13.86	4.31	14.00	13.58	13.86	4.67
Fairness Index	0.428	0.497	0.180	0.498	0.340	0.477	0.186	0.561
Mean RB occupancy (%)	100	97	99	100	100	97	99	100

## Conclusion

The LTE-A is one of the best wireless transmission platforms and would be a part of the future cellular networks. In this paper, performance of four widely used schedulers; RR, BCQI, FFR and PF has been investigated under two different scenarios considering the LTE-A environment as a case study. The working of the schedulers in the downlink LTE-A system was evaluated and compared in terms of throughput, fairness and spectral efficiency. The results have shown that BCQI algorithm outperforms the other three in terms of throughput because it is a sum rate maximizing resource allocation algorithm in which the priority of each RB assigned is according to the CQI feedback value. BCQI scheduler performs better than other schedulers for the users located within the coverage range of eNodeB with better channel conditions while the edge users with bad channel quality are rejected. It makes the implementation of BCQI in real time scenario difficult as a percentage of users in blind spots with bad channel quality would be rejected. For such users, the performance of other schedulers is much better, especially the FFR scheduler that provides a decent throughput. Similarly, the fairness index for FFR and PF is better than others for users suffering from bad channel conditions. It is evident that each scheduling algorithm has its own advantages and disadvantages, and a trade-off has to be made in the scheduler choice. On a careful consideration of all the tested parameters, the FFR scheduler appears to be the best out of the four tested. Flexibility and adaptability in the design and capability to embed other schedulers in its process further makes FFR a recommended solution for LTE-A wireless links with high QoS.

##  Supplemental Information

10.7717/peerj-cs.546/supp-1 Supplemental Information 1 Matlab code for different scheduling algorithms.

## Additional Information and Declarations

### Data Availability

The following information was supplied regarding data availability: The MATLAB code files used for different schedulers are available in the Supplemental File.
